# *Plasmodium* infection and oxidative status in breeding great tits, *Parus major*

**DOI:** 10.1186/s12936-016-1579-9

**Published:** 2016-11-04

**Authors:** Jessica Delhaye, Tania Jenkins, Philippe Christe

**Affiliations:** Department of Ecology and Evolution, University of Lausanne, 1015 Lausanne, Switzerland

**Keywords:** Antioxidants, Avian malaria, Oxidative damage, Parasitism, Pro-oxidants, Reproduction

## Abstract

**Background:**

*Plasmodium* parasites may affect the oxidative status of their hosts, defined as the balance of pro-oxidant compounds and antioxidant defences in an organism. An increased energy requirement, the activation of immune functions or the parasite itself may lead to a higher production of pro-oxidants and/or an antioxidant depletion resulting in a higher oxidative stress and associated damage in infected individuals. Relatively little is known about the mechanisms underlying oxidative processes at play during host-*Plasmodium* interaction in the wild.

**Methods:**

The effect of *Plasmodium* infection on host oxidative status was investigated in wild populations of breeding great tits, *Parus major*, naturally infected by *Plasmodium* spp. When chicks were 14 days old, the parents were blood-sampled to measure four complementary oxidative status markers: pro-oxidant production as mitochondrial superoxide production in red blood cells (RBC), antioxidant defences as plasma antioxidant capacity and oxidative damage as reactive oxygen metabolites in the plasma and RBC membrane resistance to oxidative attack.

**Results:**

*Plasmodium*-infected individuals produced more pro-oxidants compared to uninfected ones and pro-oxidant production positively correlated to infection intensity. There was also a conditional effect of reproductive effort on oxidative damage depending on *Plasmodium* infection status. There was no direct effect of infection on oxidative damage and no effect on antioxidant defences.

**Conclusions:**

The results suggest that *Plasmodium* parasites may impose a cost in terms of increased oxidative stress possibly mediated via a higher energy requirement in infected hosts. This further suggests that *Plasmodium* parasites may modify host life history traits via an induction of oxidative stress. This study highlights that measuring several complementary oxidative status markers may enable to capture oxidative processes at play during host-*Plasmodium* interactions.

**Electronic supplementary material:**

The online version of this article (doi:10.1186/s12936-016-1579-9) contains supplementary material, which is available to authorized users.

## Background

*Plasmodium* parasites, which cause malaria, are ubiquitous parasites infecting a wide range of vertebrate species [1] on which they impose fitness costs ranging from decreased survival [2–4], decreased fecundity [5] to lower levels of disease severity [6, 7]. Regardless of the direct fitness cost of parasite infection, malaria induces physiological changes in hosts, which may subsequently affect a host’s oxidative status. An organism’s oxidative status is the relative amount of pro-oxidant compounds and antioxidant defences. Pro-oxidants are generated as by-products of metabolism [8] or during particular physiological processes such as cell signalling [9, 10] or defence against parasites [11, 12]. They are highly reactive compounds that when not sufficiently balanced by antioxidant defences can react with other biomolecules generating oxidative damage to lipids, proteins and DNA [8]. This imbalance, known as oxidative stress, is harmful and results in dysfunctions at the molecular, cellular and organ level [8]. For instance, it has been linked with male infertility [13], cancer [14], chronic diseases [15], neurodegenerative diseases [16] and most notably the ageing process [17, 18]. Therefore, through their effect on host physiology, *Plasmodium* parasites may have a large impact on host life history traits.

**Fig. 1:**
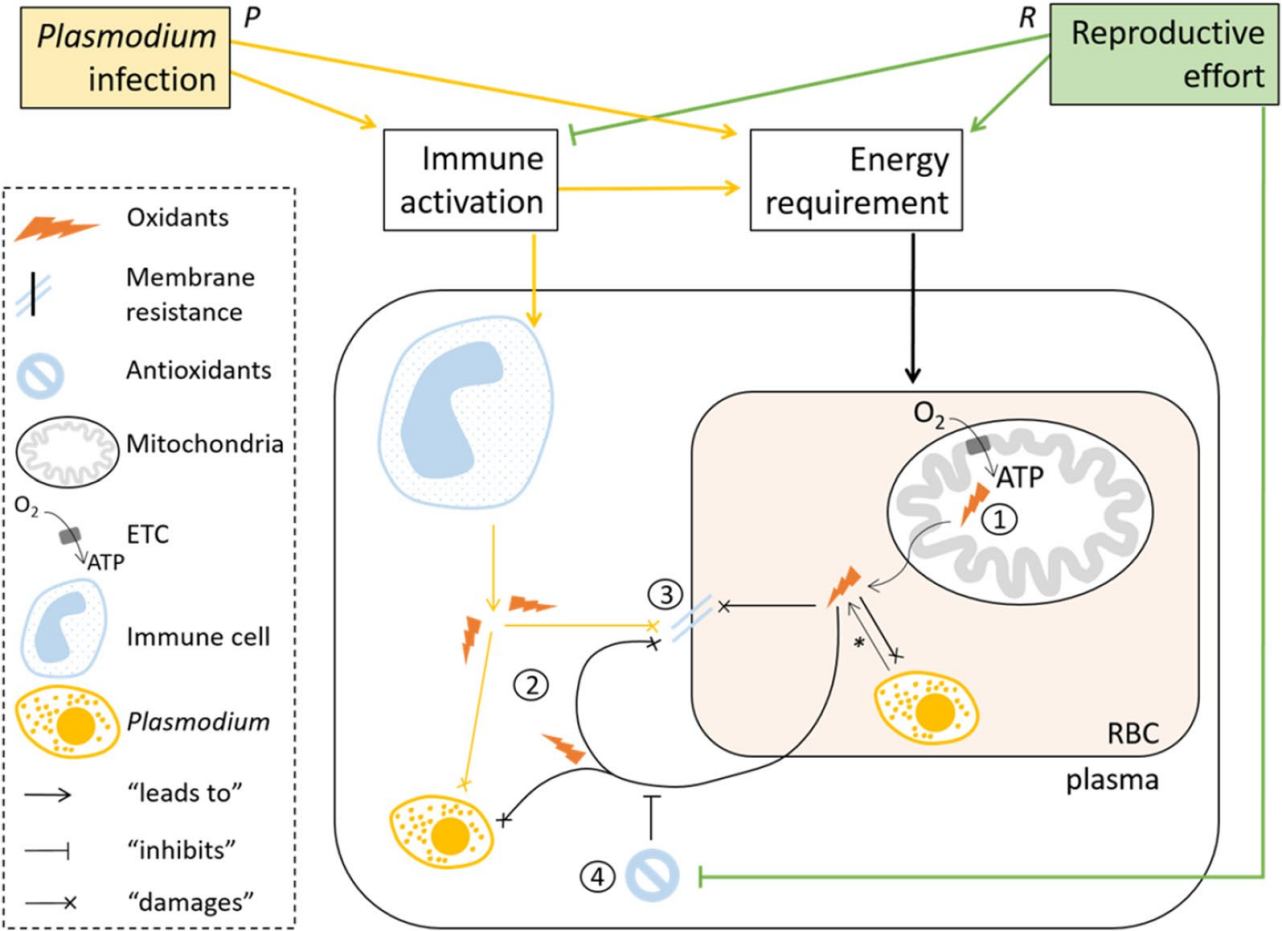
Schematic of the effect of *Plasmodium* infection and reproductive effort on bird oxidative status. The *P* (*yellow*) and *R* (*green*) pathways show how *Plasmodium* infection and reproductive effort, by requiring energy, can lead to an enhancement of superoxide production by the mitochondrial electron transport chain (ETC) during mitochondrial respiration and consumption of oxygen (O_2_) to generate adenosine triphosphate (ATP). Oxidants can increase in the red blood cell (RBC) compartment and damage intracellular stages of *Plasmodium* parasite (merozoite), damage other host’s biomolecules such as lipids of the RBC membrane, enhance plasmatic oxidants and oxidative damage by oxidative reaction chain. The *P* pathway also shows how *Plasmodium* infection, by activating the host’s immune system can increase the plasmatic oxidant level. Plasmatic oxidants produced by *R* and *P* pathways are able to damage extracellular stages of *Plasmodium* parasite (merozoite), damage other host’s biomolecules such as lipids of RBC membrane. The plasma antioxidant barrier can counteract the oxidative cascade in the plasma. Reproductive effort can also deplete antioxidant defences and inhibit immune activation. Intracellular stages of *Plasmodium* can also enhance oxidants in the RBC compartment through haemoglobin degradation. Black paths are not specific to the *P* or *R* pathways. *Circled numbers* indicate oxidative status markers measured in the present study (*1* RBC superoxide production, *2* reactive oxygen metabolites in the plasma, *3* RBC membrane resistance to oxidative attack, *4* plasma antioxidant capacity)

There are several ways by which host oxidative processes may be altered during host-*Plasmodium* interaction (*P* pathway, Fig. 1). *Plasmodium* infection may modify the energy and resource allocation of the host. Parasites may divert host resources for their own development, increasing host energy requirement and pro-oxidant production and/or depleting host antioxidant resources. Parasites may also induce sickness behaviour during which the hosts will reallocate energy/resources away from secondary activities, such as locomotion or reproduction, towards immune functions [19]. For example, infected individuals have been shown to increase their reproductive investment when they were experimentally cleared of an infection [5]. *Plasmodium* infection also induces host’s immune activation. Although discussed in the literature, immune functions can be energetically costly [20–23] and are further linked to the oxidative process. For example, immune activation [24], inflammation [25–27] and the T cell mediated immune response [28–30] partly rely on pro-oxidant production to damage invading parasites which are also sensitive to oxidative attacks. Both in vitro and in vivo experimental studies have shown that pro-oxidants inhibit *Plasmodium* development, which suggests that they are a defence mechanism against *Plasmodium* [31–33]. Pro-oxidants produced during the immune response have also been shown to lead to collateral oxidative damage to the host [25]. Finally, *Plasmodium* parasites themselves may generate pro-oxidants through the degradation of haemoglobin in infected red blood cells (RBC) [31]. As a result, *Plasmodium*-infected individuals may experience an increased pro-oxidant generation or an antioxidant depletion and suffer increased oxidative stress leading to oxidative damage. Both *Plasmodium* infection alone and infection intensity (i.e. parasitaemia) of *Plasmodium* have been previously associated with increased oxidative damage to plasma metabolites and to DNA [2, 34, 35]. Taken together, these results show that the oxidative stress phenomenon and its interplay with infection are complex and therefore need to be studied using a range of markers that capture different aspects of the oxidative process.

The effect of *Plasmodium* infection on host oxidative status can also depend on other factors, such as reproduction (*R* pathway, Fig. 1). For example, it has recently been shown that infected individuals experienced increased susceptibility to oxidative stress during reproduction, but only during the phase of young provisioning, which requires a high energy investment [36]. Reproduction can also affect oxidative status. High energy investment during reproduction has been linked with a decreased resistance to oxidative stress [37], decreased antioxidant defences [38, 39] and an increased oxidative stress [40, 41]. There can also be resource allocation trade-offs in reproductively active individuals [42] leading to a lower immunocompetence (i.e. the ability to mount an immune response) and/or higher parasitism rates [43–46]. Oxidative stress is therefore influenced by a range of factors, yet at the moment, little is known about how infection and reproduction interact to affect different aspects of the oxidative process.

Avian malaria is a popular biological system to study host-*Plasmodium* interactions in the wild and has been used as a model to study broad biological phenomena such as fitness consequences of parasite infection or host-parasite coevolution [47]. The great tit, *Parus major*, the focus of the current study, can be infected by several *Plasmodium* lineages [48–50] among which one of the most common avian malaria lineage: *Plasmodium relictum* SGS1 [1]. To date, there is no consensus on the effect of age or sex on the prevalence of infection [34, 43, 51, 52] but the infection has been shown to be highly persistent (i.e. chronic infection) across years [53]. Infection does not appear to affect body mass or condition [34, 37, 52]. In studies where reproductive effort has been manipulated the prevalence of infection as well as the parasite intensity have been shown to increase in males but not in females [37, 43]. Infection has also been associated with a reduced probability of breeding in two consecutive years in males [43] but not in females [51]. These sex differences in the susceptibility to *Plasmodium* could suggest a role for host physiology during the host-*Plasmodium* interaction.

In this study, the link between natural *Plasmodium* infection and individual oxidative status was investigated in wild populations of breeding great tits. *Plasmodium* parasite development occurs in host red blood cells (RBC) [1], a key phase for *Plasmodium* parasites to complete their life cycle and for the host to mount an immune response [54]. Oxidative status was therefore measured in the blood using four complementary oxidative status markers at the cellular and plasmatic levels (Fig. 1). Four markers were used to capture different aspects of the oxidative process: RBC superoxide production, reactive oxygen metabolites (ROMs) in the plasma, RBC membrane resistance to attack and plasma antioxidant capacity. Superoxides are the first pro-oxidant molecules released by the mitochondrial electron transport chain (ETC) during mitochondrial respiration [55]. In contrast to mammals, avian RBCs have functional mitochondria in their cytoplasm [56] and so, superoxide production may reflect individual energy consumption. Superoxides can also generate further pro-oxidant compounds through the oxidative reaction chain [55]. ROMs are oxidized molecules which may reflect oxidative processes occurring in the plasmatic compartment such as oxidation caused by immune effectors during the immune response. They also act as pro-oxidant compounds. Membrane resistance to oxidative attack reflects the amount of past oxidative attacks that have not been counteracted (i.e. oxidative damage) and the current resistance to oxidation (i.e. antioxidant defence, see [57, 58]). Infected red blood cells are characterised by rigid membranes [59], a phenomenon which could be associated with increased oxidative damage and a decreased resistance to oxidative attack. Finally, plasma contains antioxidant defences which can oppose the oxidative process of biomolecules. These circulating defences protect the free molecules and the membranes of (host and parasitic) cells present in the plasma. The bird reproductive season can be divided into two time points that are particularly energetically costly and could have an effect on oxidative status: egg laying and chick rearing reflecting an early and a later cost. Both phases were considered to investigate the interplay between infection and reproductive effort on oxidative status. Pro-oxidant production and oxidative damage were expected to be higher in infected individuals and to increase with reproductive effort. Plasma antioxidant defences were expected to be either depleted because of the oxidative processes associated with infection and reproduction or increased as a protective response against oxidative stress. In focusing on these four markers, the aim was to gain a comprehensive overview of the oxidative processes at play and to highlight which pathway leads to oxidative stress enhancement during host-*Plasmodium* interactions.

## Methods

### Great tit sampling

Three populations of great tits were monitored during the 2012 breeding season (from March to June) at three sites in Switzerland: the forests of Dorigny (46°31′N; 6°34′E; alt. 380 m), Monods (46°34′N; 6°24′E; alt. 668 m), and La Praz (46°40′N; 6°20′E; alt. 1000 m). The great tit is a species with a large geographical distribution that readily nests in artificial nest-boxes allowing a rigorous monitoring of natural populations. Nest boxes were visited periodically to estimate the date at which the first egg was laid, the clutch size (i.e. the number of incubated eggs) and the hatching date. Brood size was defined as the number of chicks surviving to 14 days. At that stage, parents were caught using door traps or mist nets. Once caught, parents were aged (<2 years old: sub-adult and ≥2 years old: adult), weighed (to the nearest 0.1 g) and blood-sampled by brachial venipuncture with sterile needles (Neolus 100, Terumo Europe) and blood was collected in lithium-heparinized microvettes (Sarstedt^®^, Germany).

### Oxidative status markers

Directly after blood sampling, 16 µl of blood were transferred into 584 µl of KRL buffer (Kirial international, Laboratoires Spiral S.A., Dijon, France), a physiological buffer adjusted to bird cell osmolarity. These diluted blood samples were stored in the dark and on ice until laboratory analyses. The blood remaining in the microvette was centrifuged for 10 min at 15,000 rcf and 4 °C. Red blood cells and plasma were split and stored separately at −20 °C. There was no effect of time elapsed between field sampling and laboratory analyses on oxidative status measurements (mean time elapsed in hours ± standard deviation: 8.94 ± 2.87; Pearson’s correlations between each measurement and time elapsed: superoxides: r = −0.01, t = −0.07, p = 0.94; ROMs: r = −0.11, t = −1.30, p = 0.19; membrane resistance: r = 0.16, t = 1.88, p = 0.06; plasma antioxidants: r = −0.05, t = −0.43, p = 0.69).

Once in the laboratory, blood cells were washed by centrifuging the diluted samples for 5 min at 500 rcf at 4 °C, and cell pellets were resuspended in a volume of 700 µl of KRL buffer. Glucose was added in excess (30 mM final concentration) as a mitochondrial respiration substrate to control for possible inter-individual variation in cell substrate availability. This stock sample solution was then used as a starting solution for the flow cytometric measurements of superoxide production and mitochondria quantity and for spectrometric measurements of RBC membrane resistance to oxidative attack.

### RBC superoxide production corrected for mitochondria quantity

Superoxide production was quantified per live RBC using the specific fluoroprobe MitoSOX Red (Molecular Probes, Invitrogen, [60]). In order to standardize superoxide production, the amount of mitochondria per RBC was also quantified using the fluorescent probe nonyl acridine orange (NAO, Molecular Probes, Invitrogen) which binds cardiolipins, phospholipids specific to the mitochondria inner membrane [61].

250 µl of stock sample solution of diluted blood was incubated for 30 min at 40 °C (i.e. ~avian body temperature) with MitoSOX Red fluorescent dye (12 µM final concentration once dye added to the sample, diluted in dimethyl sulfoxyde, DMSO). Another 250 µl of stock sample solution was incubated for 7 min at 40 °C with NAO (10 µM final concentration). At the end of the incubation period, cells were washed by centrifuging samples for 5 min at 500 rcf at 4 °C. Cells were then resuspended in 300 µl of KRL buffer and stored in the dark and on ice until flow cytometer measurement on a FACS Calibur (Becton–Dickinson) using the FL2 channel and excitation at 582 nm for superoxide production, and using the FL1 channel and excitation at 200 nm for mitochondria quantity. 50,000 events per sample were acquired and the median fluorescence value (i.e. arbitrary unit) was computed for each sample using the BD CellQuest Pro software (Becton–Dickinson, coefficient of intra-individual variation: superoxide production: CV = 2.51%; mitochondria quantity: CV = 2.14%, four individuals ran eight times).

Superoxide production (log transformed to achieve normality) and mitochondria quantity (square root transformed to achieve normality) per RBC were positively correlated (Pearson’s correlation: r = 0.52, t = 7.15, df = 140, p < 0.001, Additional file 1). In order to obtain a corrected measurement of superoxide production per mitochondria, the ratio of RBC superoxide production on RBC mitochondria quantity was calculated for each sample. In the following text, “superoxide production” refers to this corrected measurement, unless otherwise specified.

### Reactive oxygen metabolites in the plasma

ROMs were quantified in the plasma using the d-ROMs test (Diacron international, Grosseto, Italy), according to the manufacturer’s protocol (although see [62] for limitations associated with this test). In the presence of iron, reactive oxygen metabolites produce peroxyl and alkoxyl radicals (Fenton’s reaction). These radicals react directly with a chromogenic substrate. After 75 min of incubation at 40 °C, optical density was measured at 540 nm and plasma sample values were compared to values obtained from a calibrator (coefficient of intra-assay variation: CV = 2.25%; coefficient of inter-assay variation: CV = 3.00%, one control ran twice on each plate). Reactive oxygen metabolites were then expressed as the equivalent of hydroperoxide quantity in the plasma (mg H_2_O_2_ l^−1^) and was square root transformed to achieve normality.

### RBC membrane resistance to oxidative attack

Membrane resistance was measured as the time needed to haemolyse half of the RBCs as described in [58]. Cell haemolysis was quantified using a microplate reader by following the decrease of optical density at the wavelength of 540 nm and using the Kirial International processing analysis software (coefficient of intra-individual variation: CV = 2.83%, 133 individuals ran in duplicates).

### Plasma antioxidant capacity

Plasma antioxidant capacity was assessed by measuring the power of the plasmatic barrier to oppose hypochlorite (HClO) induced oxidation using the Oxy-Adsorbent test (Diacron international, Grosseto, Italy), according to the manufacturer’s protocol. After 10 min of incubation at 40 °C, HClO excess was determined with a chromogenic substrate by optical density measurement at 540 nm and plasma sample values were compared to values obtained from a calibrator (coefficient of intra-assay variation: CV = 3.82%; coefficient of inter-assay variation: CV = 5.12%, one control ran twice on each plate). The final HClO concentration was expressed in µmol ml^−1^ and was used as a proxy of plasma antioxidant capacity. Plasma antioxidant capacity was log transformed to achieve normality.

### Plasmodium detection and quantification

DNA was extracted from blood using the DNeasy blood and tissue extraction kit (Qiagen^®^, Valencia, CA, USA) according to the manufacturer’s protocol for the BioSprint 96. A nested PCR was performed to detect the infection status of individuals. Following a primary reaction with the primers HaemNF1 and HaemNR3, a secondary reaction with the primers HaemF and HaemR2 to amplify *Plasmodium* and *Haemoproteus* was conducted [63] and 5 μl of PCR product was run on a 2% agarose gel to assess infection status. PCRs were performed in duplicate, with one positive control for every 30 samples and one negative for every 15 (alternately water or an uninfected bird). To confirm the infection, the positive products were purified (Promega) and sent for sequencing in both directions using *HaemF* and *HaemR2* primers (GATC- Biotech, Germany). Sequences were analysed with BioEdit and identified with a local BLAST search against the MalAvi database [64], unless there were multiple-lineage infections, identified as double-base callings on the chromatogram. *Haemoproteus* infected individuals (1.6% of infected individuals) were discarded from the analyses to focus on *Plasmodium* infection.

For *Plasmodium* positive samples, parasite quantification was performed by quantitative PCR as described in [52]. Briefly, two separate qPCR reactions using a parasite cyt b TaqMan probe (CY3-CYTb-BHQ2) and a host 18 s rRNA probe (FAM-18S-BHQ1) were performed. For both parasite and host, DNA concentration was calculated from a standard curve and the parasitaemia was given by the ratio of the parasite DNA concentration on the host DNA concentration. Parasitaemia was log transformed to achieve normality.

### Statistical analyses

The aim was to investigate the effect of *Plasmodium* infection on oxidative status, measured using four complementary markers, and to further explore the interaction between infection and reproductive effort. All statistical analyses were performed using R (version 3.1, [65]). The following oxidative status markers were analysed as response variables in linear mixed-effects models (lme function in nlme package): (1) RBC superoxide production (corrected for mitochondria quantity) (2) reactive oxygen metabolites in the plasma, (3) RBC membrane resistance to oxidative attack, and (4) plasma antioxidant capacity. A term was included for *Plasmodium* infection status (uninfected or infected). As clutch and brood sizes were correlated (Pearson’s correlation: r = 0.43, t = 4.58, df = 90, p < 0.001), they were not included simultaneously in a model, but the same models were alternatively tested with reproductive effort as brood size or as clutch size. The two-way interaction between infection status and reproductive effort was included. Terms for body mass, age (sub-adults or adults) and sex, which are known to influence oxidative stress [66–69], as well as hatching date which may account for seasonal variation were also included. To explore potential interactions with infection, the interaction terms between infection and age and between infection and sex were also fitted. Nest box nested in site was included as a random factor. To determine the explanatory power of each fitted parameter, likelihood ratio tests were conducted following a standard backward procedure by sequential elimination from the full model. Non-significant terms (p > 0.05) were dropped out from the full model to reach the minimal adequate one [70]. Significant p values in the text come from minimal adequate models, and non-significant p values come from the likelihood ratio test before the term was dropped out. To look at the effect of each significant term individually, contrast analyses were performed [70].

For the pro-oxidant production, supplementary models, similar to the ones described above, but considering superoxide production (not corrected for mitochondria quantity) as a response variable and including mitochondria quantity as an explanatory variable were run.

The correlations between the different oxidative status markers were explored. The relationships between each marker and parasitaemia were also investigated in infected individuals only.

## Results

In total, 92 broods were monitored at the three study sites among which 44.0% of the females and 41.1% of the males caught were infected by *Plasmodium* spp. (Table 1). Half of the infected birds were carrying *Plasmodium* lineage SGS1 (55.2%). Among the rest, 15.5, 12.1, 10.3 and 1.7% of the infections were caused by the lineages BT7, TURDUS1, SW2, and GRW11 respectively. The other 5.2% were mixed infections with more than one lineage.

**Table 1:** *Plasmodium* infection prevalence

Site	% (n)	Sex	% (n)	Age	% (n)
Dorigny	40.2 (82)	Females	44.0 (75)	Sub-adults	45.1 (51)
Monods	59.5 (42)	Males	41.1 (73)	Adults	41.2 (97)
La Praz	20.8 (24)				

Prevalence (%) of *Plasmodium* infection related to the site of capture, sex and age. Sample size (n) is indicated in brackets

The following results provided in the text refer to the models considering reproductive effort as brood size and models considering reproductive effort as clutch size are provided in Additional file 2.

### RBC superoxide production

**Fig. 2:**
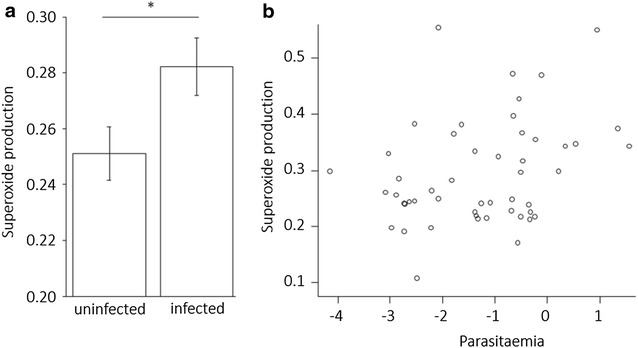
*Plasmodium* infection and pro-oxidant production. **a** Superoxide production corrected for mitochondria quantity (arbitrary unit) in uninfected and infected individuals. The *star* indicates significant difference at p < 0.05. **b** Superoxide production corrected for mitochondria quantity (arbitrary unit) in relation to parasitaemia (arbitrary unit, log transformed) in infected individuals

RBC superoxide production was higher in infected individuals compared to uninfected ones (Table 2; Fig. 2a) and increased with brood size (Table 2). It was higher in adults compared to sub-adults (mean ± standard error in sub-adults: 0.2450 ± 0.0121, in adults: 0.2884 ± 0.0087; Table 2) and in females compared to males (mean ± standard error in females: 0.2815 ± 0.0092, in males: 0.2520 ± 0.0102; Table 2). It also increased with body mass (Table 2). When considering reproductive effort as clutch size, the same minimal adequate model was obtained (Additional file 2). When analysing superoxide production not corrected for mitochondrial quantity, the effects of infection and age on superoxide production remained and superoxide production also increased with mitochondrial quantity (p < 0.001, Additional file 3). The effects of sex and body mass were not significant (Additional file 3). The effect of reproductive effort remained only in the model containing clutch size (Additional file 3). RBC superoxide production was positively correlated to parasitaemia in infected individuals (Pearson’s correlation: r = 0.33, t = 2.40, df = 48, p = 0.020, Fig. 2b).

**Table 2:** Minimal adequate models of oxidative status markers

	Estimate	SE	t value	p value
Superoxides				
*Intercept*	*0.0163*	*0.1119*	*0.15*	*0.885*
*Body mass*	*0.0143*	*0.0062*	*2.29*	*0.026*
*Age*	*0.0434*	*0.0145*	*2.99*	*0.004*
*Sex*	−*0.0295*	*0.0121*	−*2.44*	*0.019*
Hatching date				0.840
*Brood size*	*0.0084*	*0.0044*	*1.91*	*0.060*
*Infection*	*0.0311*	*0.0126*	*2.47*	*0.017*
Infection: age				0.074
Infection: sex				0.823
Infection: brood size				0.757
ROMs				
*Intercept*	*5.3231*	*1.0428*	*5.10*	<*0.001*
Body mass				0.147
*Age*	*0.9421*	*0.4480*	*2.10*	*0.041*
*Sex*	−*1.0925*	*0.3917*	−*2.79*	*0.008*
Hatching date				0.345
*Brood size*	*0.2640*	*0.1584*	*1.67*	*0.099*
*Infection*	*4.0244*	*1.5971*	*2.52*	*0.015*
Infection: age				0.122
Infection: sex				0.629
*Infection: brood size*	−*0.6147*	*0.2528*	−*2.43*	*0.019*
Membrane resistance				
*Intercept*	*95.0378*	*4.4943*	*21.15*	<*0.001*
Body mass				0.787
Age				0.919
*Sex*	*3.1481*	*0.8484*	*3.71*	<*0.001*
*Hatching date*	−*0.4560*	*0.0969*	−*4.71*	<*0.001*
*Brood size*	−*1.2989*	*0.3879*	–*3.35*	*0.001*
Infection				0.113
Infection:age				0.558
Infection: sex				0.290
Infection: brood size				0.365
Antioxidant capacity				
NULL				
Body mass				0.671
Age				0.087
Sex				0.173
Hatching date				0.336
Brood size				0.084
Infection				0.816
Infection: age				0.972
Infection: sex				0.165
Infection: brood size				0.897

The markers are superoxide production (superoxide production corrected for mitochondria quantity; *n*_*total*_ 141, *n*_*sub*-*adult/adult*_ 47/94, *n*_*female/male*_ 70/71, *n*_*uninfected/infected*_ 80/61), reactive oxygen metabolites (*ROMs* square root transformed; *n*_*total*_ 134, *n*_*sub*-*adult/adult*_ 43/91, *n*_*female/male*_ 65/69, *n*_*uninfected/infected*_ 73/61), RBC membrane resistance to oxidative attack (*n*_*total*_ 133, *n*_*sub*-*adult/adult*_ 50/83, *n*_*female/male*_ 67/66, *n*_*uninfected/infected*_ 75/58) and plasma antioxidant capacity (log transformed; *n*_*total*_ 69, *n*_*sub*-*adult/adult*_ 16/53, *n*_*female/male*_ 34/35, *n*_*uninfected/infected*_ 43/26) and the models considered reproductive effort as brood size

Minimal models are given in italic with intercept, as well as estimate, standard error (SE), t value and p value for each term. Non-significant terms that were tested, are given with the p value of the likelihood ratio test before being dropped out of the model

### Reactive oxygen metabolites in the plasma

**Fig. 3:**
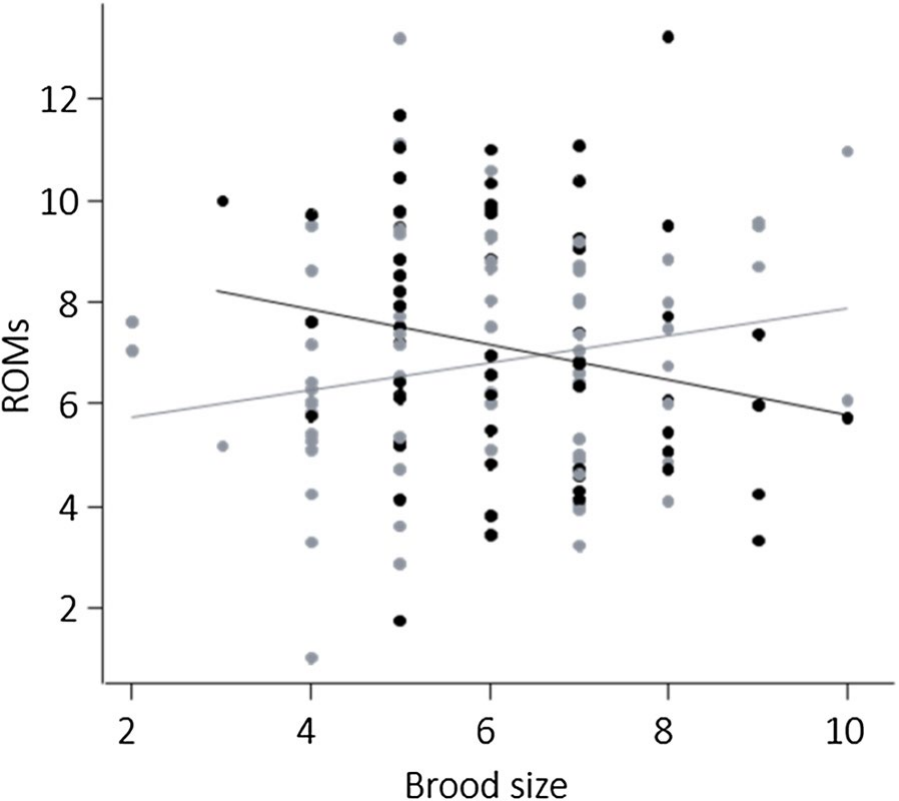
Oxidative damage as a function of reproductive effort and infection status. Reactive oxygen metabolites (ROMs, mg H_2_O_2_ l^−1^, square root transformed) in relation to brood size in uninfected (*grey circles* and *line*) and infected individuals (*black circles* and *line*)

There was no effect of infection *per se* on ROM quantity but there was a significant interaction between infection status and brood size (Table 2). The a posteriori contrast analysis showed that ROM quantity tended to increase with brood size in uninfected individuals (contrast: p = 0.099) and/or to decrease with brood size in infected ones (contrast: p = 0.090, Fig. 3). ROMs were higher in adults than in sub-adults (mean ± standard error in sub-adults: 6.5256 ± 0.3904, in adults: 7.4677 ± 0.2867; Table 2) and in females than in males (mean ± standard error in females: 7.5429 ± 0.3109, in males: 6.4504 ± 0.3381; Table 2). When considering reproductive effort as clutch size, there was no interaction between infection and reproductive effort and only the effects of age and sex remained (Additional file 2). There was no correlation between ROMs and parasitaemia (Pearson’s correlation: r = −0.15, t = −1.04, df = 48, p = 0.303).

### RBC membrane resistance to oxidative attack

Neither *Plasmodium* infection status nor its interaction with reproductive effort, age or sex had any effect on RBC membrane resistance (Table 2). Membrane resistance, however, decreased with brood size and with hatching date (Table 2) and was lower in females compared to males (mean ± standard error in females: 70.8753 ± 0.7655, in males: 74.0023 ± 0.7704; Table 2). When considering reproductive effort as clutch size, the same minimal adequate model was obtained (Additional file 2). There was no correlation between membrane resistance and parasitaemia (Pearson’s correlation: r = 0.00, t = −0.03, df = 45, p = 0.980).

### Plasma antioxidant capacity

None of the fitted parameters explained variation in plasma antioxidant capacity and no minimal adequate model was reached after model selection when considering reproductive effort measured as brood or clutch size (Table 2, Additional file 2). Plasma antioxidant capacity was not correlated to parasitaemia (Pearson’s correlation: r = −0.14, t = −0.68, df = 22, p = 0.502).

### Correlations among the different oxidative status markers

ROM quantity was significantly positively correlated to both superoxide production and plasma antioxidant capacity (Table 3). Membrane resistance tended to be negatively correlated to both superoxide production and ROM quantity (Table 3). None of the other markers were correlated to one another (Table 3).

**Table 3:** Pearson’s correlations between oxidative status markers

Correlations	r	t value	df	p value
A				
SO–ROMs	0.25	2.91	129	0.004
SO–membrane resistance	−0.16	−1.81	125	0.073
SO–antioxidant capacity	0.11	0.88	64	0.380
ROMs–membrane resistance	−0.18	−1.97	118	0.051
ROMs–antioxidant capacity	0.24	2.06	67	0.043
Membrane resistance–antioxidant capacity	−0.20	−1.53	59	0.131

The markers are: superoxide production (*SO* superoxide production corrected for mitochondria quantity, reactive oxygen metabolites (*ROMs* square root transformed), RBC membrane resistance to oxidative attack and plasma antioxidant capacity (log transformed)

Results are given with correlation coefficient (r), t value, degree of freedom (df) and p value

## Discussion

In this study, the link between natural *Plasmodium* infection and oxidative status in wild breeding great tits was explored measuring four complementary oxidative status markers. The presence and the intensity of infection were associated with an elevated production of pro-oxidants. There was a link between reproductive effort and oxidative damage that was affected by infection status. There was however no effect of *Plasmodium* infection on antioxidant defences. These results illustrate the effect of *Plasmodium* infection on bird oxidative status and the importance of considering multiple physiological markers in oxidative stress research.

### Infection and oxidative status

*Plasmodium*-infected individuals produced more RBC superoxides compared to uninfected ones. This is the first time an RBC superoxide production increase is reported in wild *Plasmodium*-infected animals and this result is in line with results from in vitro studies that have shown that there is an increase in pro-oxidant levels in *Plasmodium*-infected RBCs [31]. RBC superoxide production increased with RBC mitochondria quantity, supporting that the superoxides were released by the host mitochondria. The allometry found for RBC superoxide further suggests that RBC superoxide production reflects individual energy consumption: the bigger the individual, the more energy consumed and the higher the superoxide production. These results suggest that the higher superoxide production could be due to an increased energy requirement in infected individuals (*P* pathway, Fig. 1). This could result from the activation of immune functions which can be energetically costly [20] or from energy diversion by the parasite for its own development. Both processes could also explain the positive correlation between infection burden and superoxide production observed in infected individuals. Superoxide produced in RBCs can further generate pro-oxidant compounds from the oxidative reaction chain [55] and lead to oxidative damage. This is supported by the positive correlation between RBC superoxide and plasma ROM levels that was found, by the negative trend observed between superoxide production and RBC membrane resistance as well as between ROM quantity and membrane resistance.

There was no direct effect of infection on host antioxidant defences or oxidative damage. The fact the *Plasmodium* infection differentially affected the four oxidative status markers may be explained by the ability of organisms to regulate their oxidative stress. Pro-oxidant production at the mitochondrial level is constrained by metabolic activity at the cellular level and its regulation is complex [71]. In contrast, circulating antioxidants are partly obtained from food and can be made available when needed from storage sources, such as organs [72, 73]. Membrane resistance is partly explained by its composition in phospholipids [74]. These are also partly obtained from food and can be selectively incorporated into the biomembranes [75]. Compared to other tissues, red blood cells have a relatively high turn-over which may allow the maintenance of a resistant pool of cells. Therefore, these two traits, plasma antioxidant capacity and membrane resistance, might be more easily maintained stable under oxidative stress conditions which might explain why they were not affected by infection status. However, investing in the maintenance of the antioxidant barrier and in the repair of oxidative damage might have a cost too. Combined, these results highlight an effect of *Plasmodium* infection on host oxidative status in line with recent work [34]. They further suggest that *Plasmodium* parasites may enhance host oxidative stress through a higher energy requirement in infected individuals.

### Reproductive effort and oxidative status

Several studies have found that reproduction is associated with antioxidant depletion and increased oxidative damage [37, 38, 68, 76], suggesting that oxidative stress is a physiological cost for breeding individuals. Consistent with these studies, we showed that reproductive effort positively correlated to pro-oxidant production and to oxidative damage measured as RBC membrane resistance, suggesting a higher oxidative stress in individuals investing more in their reproduction (*R* pathway, Fig. 1). There were also sex differences in oxidative status with females producing more pro-oxidants and accumulating more damage (both ROMs and membrane resistance) than males, consistent with previous study that have shown a higher oxidative stress in females than in males during reproduction [68]. The sex difference in susceptibility to oxidative stress has been suggested to stem from physiological (e.g. hormones) and activity (e.g. parental tasks) differences between sexes during reproduction [68]. Finally, there was a conditional effect of reproductive effort (measured as brood size) on oxidative damage (measured as ROMs) depending on the infection status, but no effect of either reproductive effort or infection status when considering reproductive effort as clutch size. A previous study on the Seychelles warblers, *Acrocephalus sechellensis,* [36] already reported increased oxidative damage in *Plasmodium*-infected individuals during the energetically costly phase of chick provisioning. In the present study, the results suggest that oxidative damage increased with reproductive effort in uninfected individuals only. However, the a posteriori contrast analysis did not allow to conclude whether ROMs increased with reproductive effort in uninfected individuals, ROMs decreased with reproductive effort in infected individuals or both. Nevertheless, the interaction suggests that the effect of infection and reproductive effort on oxidative status are not independent or simply additive. For instance, infected individuals could have a higher investment in antioxidant defences (other than those measured in this study) as a response to infection, which would confer them a greater protection from reproduction-induced oxidative stress. It is clear that the oxidative stress phenomenon and the interplay between *Plasmodium* infection and reproductive effort on host oxidative status are complex.

### Age and oxidative status

It is well known that older individuals may experience a higher oxidative stress compared to younger ones [66, 67]. This has been partly attributed to accumulated damage to somatic cells, telomere degradation and progressive dysfunctions of metabolic pathways involved in oxidative stress regulation [17, 18]. Consistent with this result, in this study, adult birds had higher pro-oxidant production and oxidative damage (ROMs). Furthermore, a recent study in the great reed warbler, *Acrocephalus arundinaceus,* showed that chronic malaria infection was linked to faster telomere degradation [2] and subsequently faster ageing in *Plasmodium*-infected individuals. The increased RBC superoxide production in infected individuals could therefore be a mechanism explaining telomere reduction previously observed in blood samples of *Plasmodium*-infected individuals [2].

## Conclusion

The present study illustrates how *Plasmodium* infection may affect host oxidative status in wild breeding individuals. The higher pro-oxidant release by mitochondria in infected individuals suggests that *Plasmodium* parasites impose a cost on their host in terms of an enhanced energy requirement resulting in increased oxidative stress. This may have further important consequences in terms of oxidative damage leading to telomere shortening in red blood cells [2] but also in other tissues [35] potentially triggering a faster ageing associated with malaria disease.

## Additional files

**Additional file 1.** Superoxide production in relation to mitochondria quantity. Superoxide production per red blood cell (arbitrary unit, log transformed) in relation to mitochondria quantity per red blood cell (arbitrary unit, square root transformed).

**Additional file 2.** Minimal adequate models of oxidative status markers. The markers are i) superoxide production (superoxide production corrected for mitochondria quantity; n_total_ = 141, n_sub-adult/adult_ = 47/94, n_female/male_ = 70/71, n_uninfected/infected_ = 80/61), ii) reactive oxygen metabolites (ROMs, square root transformed; n_total_ = 134, n_sub-adult/adult_ = 43/91, n_female/male_ = 65/69, n_uninfected/infected_ = 73/61), iii) RBC membrane resistance to oxidative attack (n_total_ = 133, n_sub-adult/adult_ = 50/83, n_female/male_ = 67/66, n_uninfected/infected_ = 75/58) and iv) plasma antioxidant capacity (log transformed; n_total_ = 69, n_sub-adult/adult_ = 16/53, n_female/male_ = 34/35, n_uninfected/infected_ = 43/26) and the models considered reproductive effort as clutch size. Minimal models are given in bold with intercept, as well as estimate, standard error (se), t value and p value for each term. Non-significant terms that were tested, are given with the p value of the likelihood ratio test before being dropped out of the model.

**Additional file 3.** Minimal adequate models of superoxide production not corrected for mitochondria quantity. RBC superoxide production was log transformed and RBC mitochondria quantity was square root transformed (n_total_ = 141, n_sub-adult/adult_ = 47/94, n_female/male_ = 70/71, n_uninfected/infected_ = 80/61). The models considered reproductive effort as A: brood size and B: clutch size. Minimal models are given in bold with intercept, as well as estimate, standard error (se), t-value and p-value for each term. Non-significant terms that were tested, are given with the p-value of the likelihood ratio test before being dropped out of the model.

## References

[1] Valkiunas G (2005). Avian malaria parasites and other haemosporidia.

[2] Asghar M, Hasselquist D, Hansson B, Zehtindjiev P, Westerdahl H, Bensch S (2015). Hidden costs of infection: chronic malaria accelerates telomere degradation and senescence in wild birds. Science.

[3] Møller AP, Nielsen JT (2007). Malaria and risk of predation: a comparative study of birds. Ecology.

[4] Atkinson CT, Saili KS, Utzurrum RB, Jarvi SI (2013). Experimental evidence for evolved tolerance to avian malaria in a wild population of low elevation Hawai’i’Amakihi (*Hemignathus virens*). EcoHealth.

[5] Knowles SCL, Palinauskas V, Sheldon BC (2010). Chronic malaria infections increase family inequalities and reduce parental fitness: experimental evidence from a wild bird population. J Evol Biol.

[6] World Health Organization (2014). Severe malaria. Trop Med Int Health..

[7] Bartoloni A, Zammarchi L (2012). Clinical aspects of uncomplicated and severe malaria. Mediterr J Hematol Infect Dis..

[8] Halliwell B, Gutteridge J (2007). Free Radicals in Biology and Medicine.

[9] Apel K, Hirt H (2004). Reactive oxygen species: metabolism, oxidative stress, and signal transduction. Annu Rev Plant Biol.

[10] Finkel T (2011). Signal transduction by reactive oxygen species. J Cell Biol.

[11] Wink DA, Hines HB, Cheng RYS, Switzer CH, Flores-Santana W, Vitek MP (2011). Nitric oxide and redox mechanisms in the immune response. J Leukoc Biol.

[12] Nathan C, Cunningham-Bussel A (2013). Beyond oxidative stress: an immunologist’s guide to reactive oxygen species. Nat Rev Immunol.

[13] Tremellen K (2008). Oxidative stress and male infertility—a clinical perspective. Hum Reprod Update..

[14] Waris G, Ahsan H (2006). Reactive oxygen species: role in the development of cancer and various chronic conditions. J Carcinog..

[15] Lambeth JD (2007). Nox enzymes, ROS, and chronic disease: an example of antagonistic pleiotropy. Free Radic Biol Med.

[16] Federico A, Cardaioli E, Da Pozzo P, Formichi P, Gallus GN, Radi E (2012). Mitochondria, oxidative stress and neurodegeneration. J Neurol Sci.

[17] Perez-Campo R, López-Torres M, Cadenas S, Rojas C, Barja G (1998). The rate of free radical production as a determinant of the rate of aging: evidence from the comparative approach. J Comp Physiol B, Biochem Syst Environ Physiol..

[18] Finkel T, Holbrook NJ (2000). Oxidants, oxidative stress and the biology of ageing. Nature.

[19] Adelman JS, Martin LB (2009). Vertebrate sickness behaviors: adaptive and integrated neuroendocrine immune responses. Integr Comp Biol.

[20] Lochmiller RL, Deerenberg C (2000). Trade-offs in evolutionary immunology: just what is the cost of immunity?. Oikos.

[21] Demas GE, Chefer V, Talan MI, Nelson RJ (1997). Metabolic costs of mounting an antigen-stimulated immune response in adult and aged C57BL/6 J mice. Am J Physiol.

[22] Freitak D, Ots I, Vanatoa A, Hõrak P (2003). Immune response is energetically costly in white cabbage butterfly pupae. Proc R Soc B Biol Sci..

[23] Martin LB, Scheuerlein A, Wikelski M (2003). Immune activity elevates energy expenditure of house sparrows: a link between direct and indirect costs?. Proc R Soc B Biol Sci..

[24] Hasselquist D, Nilsson J-Å (2012). Physiological mechanisms mediating costs of immune responses: what can we learn from studies of birds?. Anim Behav.

[25] Bertrand S, Criscuolo F, Faivre B, Sorci G (2006). Immune activation increases susceptibility to oxidative tissue damage in Zebra Finches. Funct Ecol.

[26] Sorci G, Faivre B (2009). Inflammation and oxidative stress in vertebrate host-parasite systems. Philos Trans R Soc Lond B Biol Sci.

[27] Sadd BM, Siva-Jothy MT (2006). Self-harm caused by an insect’s innate immunity. Proc R Soc B Biol Sci..

[28] Costantini D, Dell’Omo G (2006). Effects of T-cell-mediated immune response on avian oxidative stress. Comp Biochem Physiol A Mol Integr Physiol..

[29] Finney OC, Riley EM, Walther M (2010). Regulatory T cells in malaria—friend or foe?. Trends Immunol.

[30] Hansen DS, Schofield L (2010). Natural regulatory T cells in malaria: host or parasite allies?. PLoS Pathog.

[31] Postma NS, Mommers EC, Eling WMC, Zuidema J (1996). Oxidative stress in malaria; implications for prevention and therapy. Pharm World Sci.

[32] Schirmer RH, Schöllhammer T, Eisenbrand G, Krauth-Siegel RL (1987). Oxidative stress as a defense mechanism against parasitic infections. Free Radic Res Commun..

[33] Bichet C, Cornet S, Larcombe S, Sorci G (2012). Experimental inhibition of nitric oxide increases *Plasmodium relictum* (lineage SGS1) parasitaemia. Exp Parasitol.

[34] Isaksson C, Sepil I, Baramidze V, Sheldon BC (2013). Explaining variance of avian malaria infection in the wild: the importance of host density, habitat, individual life-history and oxidative stress. BMC Ecol.

[35] Asghar M, Palinauskas V, Zaghdoubi-Allan N, Valkiunas G, Mukhin A, Platonova E (1836). Parallel telomere shortening in multiple body tissues owing to malaria infection. Proc R Soc B Biol Sci..

[36] van de Crommenacker J, Richardson DS, Koltz AM, Hutchings K, Komdeur J (2012). Parasitic infection and oxidative status are associated and vary with breeding activity in the Seychelles warbler. Proc R Soc B Biol Sci..

[37] Christe P, Glaizot O, Strepparava N, Devevey G, Fumagalli L (2012). Twofold cost of reproduction: an increase in parental effort leads to higher malarial parasitaemia and to a decrease in resistance to oxidative stress. Proc R Soc B Biol Sci..

[38] Alonso-Alvarez C, Bertrand S, Devevey G, Prost J, Faivre B, Sorci G (2004). Increased susceptibility to oxidative stress as a proximate cost of reproduction. Ecol Lett.

[39] Wiersma P, Selman C, Speakman JR, Verhulst S (2004). Birds sacrifice oxidative protection for reproduction. Proc R Soc B Biol Sci..

[40] Fletcher QE, Selman C, Boutin S, McAdam AG, Woods SB, Seo AY (2012). Oxidative damage increases with reproductive energy expenditure and is reduced by food-supplementation. Evolution.

[41] Guindre-Parker S, Baldo S, Gilchrist HG, Macdonald CA, Harris CM, Love OP (2013). The oxidative costs of territory quality and offspring provisioning. J Evol Biol.

[42] Stearns SC (1992). The evolution of life histories.

[43] Richner H, Christe P, Oppliger A (1995). Paternal investment affects prevalence of malaria. Proc Natl Acad Sci USA.

[44] Ilmonen P, Hasselquist D, Langefors A, Wiehn J (2003). Stress, immunocompetence and leukocyte profiles of pied flycatchers in relation to brood size manipulation. Oecologia.

[45] Tomás G, Merino S, Moreno J, Morales J, Martínez-De La Puente J (2007). Impact of blood parasites on immunoglobulin level and parental effort: a medication field experiment on a wild passerine. Funct Ecol.

[46] Knowles SCL, Nakagawa S, Sheldon BC (2009). Elevated reproductive effort increases blood parasitaemia and decreases immune function in birds: a meta-regression approach. Funct Ecol.

[47] Marzal A, Okwa OO (2012). Recent advances in studies on avian malaria parasites. malaria parasites.

[48] Glaizot O, Fumagalli L, Iritano K, Lalubin F, Van Rooyen J, Christe P (2012). High prevalence and lineage diversity of avian malaria in wild populations of great tits (*Parus major*) and mosquitoes (*Culex pipiens*). PLoS ONE.

[49] Mata VA, da Silva LP, Lopes RJ, Drovetski SV (2015). The Strait of Gibraltar poses an effective barrier to host-specialised but not to host-generalised lineages of avian Haemosporidia. Int J Parasitol.

[50] van Rooyen J, Lalubin F, Glaizot O, Christe P (2013). Altitudinal variation in haemosporidian parasite distribution in great tit populations. Parasit Vectors..

[51] Oppliger A, Christe P, Richner H (1997). Clutch size and malarial parasites in female great tits. Behav Ecol.

[52] Jenkins T, Delhaye J, Christe P (2015). Testing local adaptation in a natural great tit-malaria system: an experimental approach. PLoS ONE.

[53] van Rooyen J, Lalubin F, Glaizot O, Christe P (2013). Avian haemosporidian persistence and co-infection in great tits at the individual level. Malar J..

[54] Taylor-Robinson AW (2010). Regulation of immunity to Plasmodium: implications from mouse models for blood stage malaria vaccine design. Exp Parasitol.

[55] Brand MD, Affourtit C, Esteves TC, Green K, Lambert AJ, Miwa S (2004). Mitochondrial superoxide: production, biological effects, and activation of uncoupling proteins. Free Radic Biol Med.

[56] Stier A, Bize P, Schull Q, Zoll J, Singh F, Geny B, Gros F, Royer C, Massemin S, Criscuolo F (2013). Avian erythrocytes have functional mitochondria, opening novel perspectives for birds as animal models in the study of ageing. Front Zool..

[57] Alonso-Alvarez C, Bertrand S, Devevey G, Prost J, Faivre B, Chastel O (2006). An experimental manipulation of life-history trajectories and resistance to oxidative stress. Evolution.

[58] Bize P, Devevey G, Monaghan P, Doligez B, Christe P (2008). Fecundity and survival in relation to resistance to oxidative stress in a free-living bird. Ecology.

[59] Paulitschke M, Nash G (1993). Membrane rigidity of red blood cells parasitized by different strains of *Plasmodium falciparum*. J Lab Clin Med.

[60] Mukhopadhyay P, Rajesh M, Yoshihiro K, Haskó G, Pacher P (2007). Simple quantitative detection of mitochondrial superoxide production in live cells. Biochem Biophys Res Commun..

[61] Mileykovskaya E, Dowhan W (2009). Cardiolipin membrane domains in prokaryotes and eukaryotes. Biochim Biophys Acta.

[62] Kilk K, Meitern R, Härmson O, Soomets U, Hõrak P (2014). Assessment of oxidative stress in serum by d-ROMs test. Free Radic Res..

[63] Hellgren O, Waldenström J, Bensch S (2004). A new PCR assay for simultaneus studies of Leucocytozoon, Plasmodium, and Haemoproteus from avian blood. J Parasitol.

[64] Bensch S, Hellgren O, Pérez-Tris J (2009). MalAvi: a public database of malaria parasites and related haemosporidians in avian hosts based on mitochondrial cytochrome b lineages. Mol Ecol Resour..

[65] R Development Core Team. R: A language and environment for statistical computing. R Foundation for Statistical Computing, Vienna, Austria. 2011. http://www.R-project.org/.

[66] Bize P, Cotting S, Devevey G, van Rooyen J, Lalubin F, Glaizot O, Christe P (2014). Senescence in cell oxidative status in two bird species with contrasting life expectancy. Oecologia.

[67] Alonso-Alvarez C, Pérez-Rodríguez L, García JT, Viñuela J, Mateo R (2010). Age and breeding effort as sources of individual variability in oxidative stress markers in a bird species. Physiol Biochem Zool.

[68] Rubolini D, Colombo G, Ambrosini R, Caprioli M, Clerici M, Colombo R (2012). Sex-related effects of reproduction on biomarkers of oxidative damage in free-living barn swallows (*Hirundo rustica*). PLoS ONE.

[69] Marko G, Costantini D, Michl G, Torok J (2011). Oxidative damage and plasma antioxidant capacity in relation to body size, age, male sexual traits and female reproductive performance in the collared flycatcher (*Ficedula albicollis*). J Comp Physiol B.

[70] Crawley MJ (2007). The R Book.

[71] Rigoulet M, Yoboue ED, Devin A (2011). Mitochondrial ROS generation and its regulation: mechanisms involved in H_2_O_2_ signaling. Antioxid Redox Signal.

[72] Beaulieu M, Schaefer HM (2013). Rethinking the role of dietary antioxidants through the lens of self-medication. Anim Behav.

[73] Elsayed NM (2001). Antioxidant mobilization in response to oxidative stress: a dynamic environmental-nutritional interaction. Nutrition..

[74] Pamplona R, Barja G, Portero-Otín M (2002). Membrane fatty acid unsaturation, protection against oxidative stress and maximum life span. A homeoviscous-longevity adaptation?. Ann N Y Acad Sci.

[75] Buttemer WA, Battam H, Hulbert AJ (2008). Fowl play and the price of petrel: long-living Procellariiformes have peroxidation-resistant membrane composition compared with short-living Galliformes. Biol Lett.

[76] Costantini D, Bonisoli-Alquati A, Rubolini D, Caprioli M, Ambrosini R, Romano M (2014). Nestling rearing is antioxidant demanding in female barn swallows (*Hirundo rustica*). Naturwissenschaften.

