# Supplementary figures and images for: Plasmodium infection and oxidative status in breeding great tits, Parus major
Source: Malar J. 2016 Nov 4;15:531. doi: 10.1186/s12936-016-1579-9 (PMC5096287; doi:10.1186/s12936-016-1579-9)

**Additional file 1.**

**
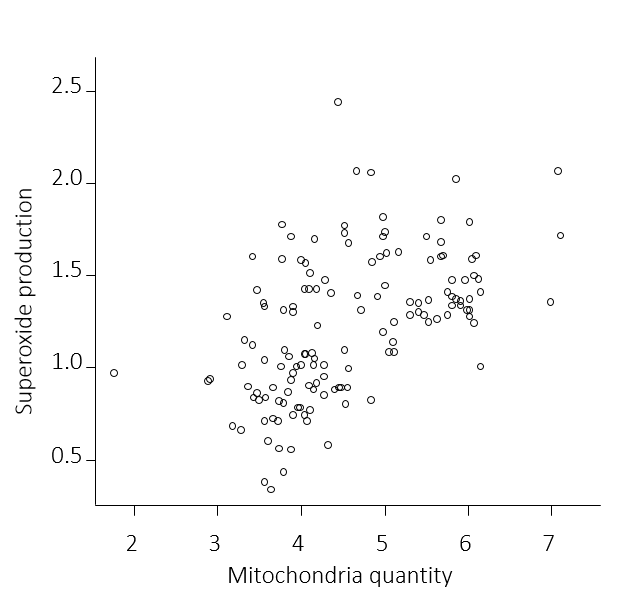
**

Supplement: Supplementary file 1 — Additional file 1. Superoxide production in relation to mitochondria quantity. Superoxide production per red blood cell (arbitrary unit, log transformed) in relation to mitochondria quantity per red blood cell (arbitrary unit, square root transformed). [file 12936_2016_1579_MOESM1_ESM.docx]
