# Supplementary material for: Plasmodium infection and oxidative status in breeding great tits, Parus major
Source: Malar J. 2016 Nov 4;15:531. doi: 10.1186/s12936-016-1579-9 (PMC5096287; doi:10.1186/s12936-016-1579-9)
Supplement: Supplementary file 2 — Additional file 2. Minimal adequate models of oxidative status markers. The markers are i) superoxide production (superoxide production corrected for mitochondria quantity; ntotal = 141, nsub-adult/adult = 47/94, nfemale/male = 70/71, nuninfected/infected = 80/61), ii) reactive oxygen metabolites (ROMs, square root transformed; ntotal = 134, nsub-adult/adult = 43/91, nfemale/male = 65/69, nuninfected/infected = 73/61), iii) RBC membrane resistance to oxidative attack (ntotal = 133, nsub-adult/adult = 50/83, nfemale/male = 67/66, nuninfected/infected = 75/58) and iv) plasma antioxidant capacity (log transformed; ntotal = 69, nsub-adult/adult = 16/53, nfemale/male = 34/35, nuninfected/infected = 43/26) and the models considered reproductive effort as clutch size. Minimal models are given in bold with intercept, as well as estimate, standard error (se), t value and p value for each term. Non-significant terms that were tested, are given with the p value of the likelihood ratio test before being dropped out of the model. [file 12936_2016_1579_MOESM2_ESM.docx]

**Additional file 2.**

| i) Superoxide production | | Estimate | se | t-value | p-value |
| --- | --- | --- | --- | --- | --- |
|  | **Intercept** | **-0.0364** | **0.1141** | **-0.32** | **0.750** |
|  | **Body mass** | **0.0150** | **0.0062** | **2.43** | **0.019** |
|  | **Age** | **0.0420** | **0.0143** | **2.94** | **0.005** |
|  | **Sex** | **-0.0292** | **0.0119** | **-2.45** | **0.018** |
|  | Hatching date |  |  |  | 0.865 |
|  | **Clutch size** | **0.0112** | **0.0042** | **2.68** | **0.009** |
|  | **Infection** | **0.0283** | **0.0125** | **2.26** | **0.028** |
|  | Infection:Age |  |  |  | 0.069 |
|  | Infection:Sex |  |  |  | 0.793 |
|  | Infection:Clutch size |  |  |  | 0.315 |
| ii) ROMs | | Estimate | se | t-value | p-value |
|  | **Intercept** | **7.9259** | **0.3528** | **22.47** | **<0.001** |
|  | Body mass |  |  |  | 0.170 |
|  | **Age** | **0.9423** | **0.4471** | **2.11** | **0.041** |
|  | **Sex** | **-1.1063** | **0.4003** | **-2.76** | **0.008** |
|  | Hatching date |  |  |  | 0.129 |
|  | Clutch size |  |  |  | 0.105 |
|  | Infection |  |  |  | 0.931 |
|  | Infection:Age |  |  |  | 0.224 |
|  | Infection:Sex |  |  |  | 0.498 |
|  | Infection:Clutch size |  |  |  | 0.131 |
| iii) Membrane resistance | | Estimate | se | t-value | p-value |
|  | **Intercept** | **91.8959** | **4.6680** | **19.69** | **<0.001** |
|  | Body mass |  |  |  | 0.830 |
|  | Age |  |  |  | 0.679 |
|  | **Sex** | **3.0810** | **0.8549** | **3.60** | **<0.001** |
|  | **Hatching date** | **-0.4082** | **0.0996** | **-4.10** | **<0.001** |
|  | **Clutch size** | **-0.8064** | **0.3766** | **-2.14** | **0.035** |
|  | Infection |  |  |  | 0.161 |
|  | Infection:Age |  |  |  | 0.657 |
|  | Infection:Sex |  |  |  | 0.356 |
|  | Infection:Clutch size |  |  |  | 0.689 |
| iv) Antioxidant capacity | | Estimate | se | t-value | p-value |
|  | NULL |  |  |  |  |
|  | Body mass |  |  |  | 0.681 |
|  | Age |  |  |  | 0.091 |
|  | Sex |  |  |  | 0.242 |
|  | Hatching date |  |  |  | 0.403 |
|  | Clutch size |  |  |  | 0.689 |
|  | Infection |  |  |  | 0.840 |
|  | Infection:Age |  |  |  | 0.911 |
|  | Infection:Sex |  |  |  | 0.079 |
|  | Infection:Clutch size |  |  |  | 0.731 |
