# Supplementary material for: Plasmodium infection and oxidative status in breeding great tits, Parus major
Source: Malar J. 2016 Nov 4;15:531. doi: 10.1186/s12936-016-1579-9 (PMC5096287; doi:10.1186/s12936-016-1579-9)
Supplement: Supplementary file 3 — Additional file 3. Minimal adequate models of superoxide production not corrected for mitochondria quantity. RBC superoxide production was log transformed and RBC mitochondria quantity was square root transformed (ntotal = 141, nsub-adult/adult = 47/94, nfemale/male = 70/71, nuninfected/infected = 80/61). The models considered reproductive effort as A: brood size and B: clutch size. Minimal models are given in bold with intercept, as well as estimate, standard error (se), t-value and p-value for each term. Non-significant terms that were tested, are given with the p-value of the likelihood ratio test before being dropped out of the model. [file 12936_2016_1579_MOESM3_ESM.docx]

**Additional file 3.**

| A | | Estimate | se | t-value | p-value |
| --- | --- | --- | --- | --- | --- |
|  | **Intercept** | **0.2805** | **0.1364** | **2.06** | **0.043** |
|  | **Mitochondria quantity** | **0.1728** | **0.0278** | **6.23** | **<0.001** |
|  | Body mass |  |  |  | 0.082 |
|  | **Age** | **0.1275** | **0.0514** | **2.48** | **0.016** |
|  | Sex |  |  |  | 0.133 |
|  | Hatching date |  |  |  | 0.119 |
|  | Brood size |  |  |  | 0.106 |
|  | **Infection** | **0.1554** | **0.0502** | **3.09** | **0.003** |
|  | Infection:Age |  |  |  | 0.103 |
|  | Infection:Sex |  |  |  | 0.990 |
|  | Infection:Brood size |  |  |  | 0.862 |
| B | | Estimate | se | t-value | p-value |
|  | **Intercept** | **-0.0558** | **0.2001** | **-0.28** | **0.781** |
|  | **Mitochondria quantity** | **0.1776** | **0.0274** | **6.48** | **<0.001** |
|  | Body mass |  |  |  | 0.065 |
|  | **Age** | **0.1338** | **0.0508** | **2.63** | **0.011** |
|  | Sex |  |  |  | 0.112 |
|  | Hatching date |  |  |  | 0.076 |
|  | **Clutch size** | **0.0394** | **0.0176** | **2.23** | **0.028** |
|  | **Infection** | **0.1404** | **0.0501** | **2.80** | **0.007** |
|  | Infection:Age |  |  |  | 0.100 |
|  | Infection:Sex |  |  |  | 0.953 |
|  | Infection:Clutch size |  |  |  | 0.743 |
